# OPTC-4. Bioinformatic analysis of COLXIα1 gene expression and its alternative splicing regulation in Paediatric Diffuse Intrinsic Pontine Gliomas (DIPGs)

**DOI:** 10.1093/noajnl/vdab070.025

**Published:** 2021-07-05

**Authors:** Malwina Jedrysik, Katie L Loveson, Nikita Kozusko, Poonam Singh, Kieren Allison, Helen L Fillmore

**Affiliations:** 1 University of Portsmouth, Portsmouth, UK; 2 Addenbrooke’s Hospital, Cambridge, UK

## Abstract

Paediatric Diffuse Intrinsic Pontine Glioma (DIPGs), is a rare aggressive childhood malignancy that arise in a region and age specific manner, with no cure and children seldom survive 2 years after diagnosis. There has been significant advances in genomic and molecular identification of driver genes as well as signature mutations that help with diagnosis. A multi-methodological approach to begin to profile the DIPG/host landscape in the context of developing brainstem was conducted. Bioinformatic analyses of a DIPG dataset and normal developing brain dataset to help separate expression associated with development from tumour was conducted. In parallel; fixed-formalin paraffin embedded DIPG tissue was obtained from post-mortem brain for focused RNA arrays and RNAseq. Two of several overlapping genes that were overexpressed in DIPG was tenascin C and the collagen XI (α1) gene (COL11A1). A common feature of these genes is that they are alternatively spliced. Therefore, the aim of this study was to focus on COL11A1, further explore its expression in diffuse midline gliomas (DMGs), to determine association with H3K27M alterations and to explore the use of bioinformatic programmes that could potentially predict the specific Col11α1 isoform expression in DIPG. The following programs were used to determine if a bioinformation approach could aid in our understanding of alternative splicing in development and DIPG: R2*: Genomics Analysis and Visualization Platform, SnapGene program, Basic Local Alignment Search Tools (BLAST), RNA Interactome Database (RNAInter). In silico analysis highlited* an RNA-binding protein *eIF4A3 as a candidate of alternative splicing regulator of* COL11A1 gene. EIF4A3 gene was shown to be upreguated in DIPG using publicly available datasets. Results shows that isoform A is the most likely isoform present in DIPG. The data obtained will help inform future *in vitro* and *in vivo* investigations into the potential role of COL11A1 and its isoforms in DIPG.

